# Parabens, Triclosan and Bisphenol A in Surface Waters and Sediments of Baiyang Lake, China: Occurrence, Distribution, and Potential Risk Assessment

**DOI:** 10.3390/toxics12010031

**Published:** 2023-12-31

**Authors:** Liguo Fu, Yaxue Sun, Jingbo Zhou, Hongbo Li, Shu-xuan Liang

**Affiliations:** 1 College of Chemistry and Materials Science, Hebei University, Baoding 071002, China; fulg628@126.com (L.F.); hbusunyaxue@163.com (Y.S.); 2 Key Laboratory of Analytical Science and Technology of Hebei Province, Baoding 071002, China; 3 Baiyangdian Basin Eco-Environmental Support Center, Shijiazhuang 050056, China; zhappygirl.8312@163.com (J.Z.);

**Keywords:** endocrine-disrupting compounds, Baiyang Lake, occurrence, spatial distribution, risk assessment

## Abstract

The extensive use of the parabens triclosan (TCS) and bisphenol A (BPA) has potential adverse effects on human health and aquatic organisms. However, their monitoring information in freshwater lakes is still limited. This study simultaneously summarized the concentrations, spatial distribution characteristics, and correlations of four types of parabens, TCS, and BPA in the surface water and sediment of Baiyang Lake. Finally, the potential risks of target pollutants were evaluated from two aspects: human health risks and ecological risks. The average contaminations of target compounds in surface water and sediment—BPA, TCS, and ∑_4_ parabens—was 33.1, 26.1, 0.7 ng/L and 24.5, 32.5, 2.5 ng/g, respectively. The total concentration of target compounds at the inlet of the upstream Fu River and Baigouyin River is significantly higher than that near Hunan and the outlet. In addition, Spearman’s correlation analysis showed a significant positive correlation between compounds. The health hazards of target compounds in surface water were all within safe limits. However, the risk quotient results indicate that in some locations in surface water, TCS poses a high risk to algae and a moderate risk to invertebrates and fish, and appropriate attention should be paid to these areas.

## 1. Introduction

Parabens, triclosan (TCS) and bisphenol A (BPA) are endocrine-disrupting compounds [1] that are widely used in many pharmaceuticals, personal care products, and industrial products, including cosmetics, beverage containers, soaps, toothpastes, plastics, food, detergents, building materials, and so on [2,3]. Compared with traditional persistent organic pollutants, these compounds have relatively short half-lives, and they are susceptible to being degraded in the environment, showing false persistence. Although the World Health Organization emphasized concerns about potential ecological impacts related to synthetic endocrine disruptors back in 2013, they have not been given sufficient attention in many countries [4]. The increased use of pharmaceutical and personal care products during the COVID-19 pandemic since early 2020 may have caused additional environmental pressure [3]. Therefore, the impact of these compounds on the environment cannot be ignored. In recent years, these compounds have been frequently reported in aquatic environments around the world, such as rivers [2,5], groundwater [6], sewage [7], lakes [3,8], oceans [9] and wetlands [10,11].

Continuous exposure to parabens TCS and BPA has been associated with adverse human health outcomes [12]. Even low concentrations of these compounds may have adverse effects on aquatic organisms [13]. Studies have suggested that parabens can interfere with the endocrine system of humans and animals [2]. TCS may form new metabolites (such as methyltrichlorosan, dioxins, etc.) with greater toxicity under the strong oxidation conditions [3,7]. High concentrations of TCS have a stimulating effect on peripheral organisms and can lead to disturbances of zebrafish [3]. BPA is a common environmental toxin with multiple toxic effects, such as reproductive toxicity, neurotoxicity, and metabolic toxicity [14]. According to reports, the occurrence and development of ovarian cancer are closely related to BPA exposure [15].

In recent years, it was proposed that manufacturers should gradually stop using these compounds in their products that come into contact with food [16]. However, a large number of other products containing these compounds are still produced and used. Due to the direct discharge of these pollutants into the environment or insufficient removal in wastewater treatment plants, water bodies are often contaminated by these compounds [17]. Therefore, understanding the occurrence and fate of these compounds in lakes is of great significance for maintaining aquatic ecosystems and human health. However, no relevant report on the occurrence of parabens in freshwater lakes around China has been published, resulting in a lack of information on the occurrence of parabens and environmental risks. Compared with parabens, the monitoring data of TCS and BPA in lakes are relatively abundant. Some researchers have investigated the distribution of TCS and BPA in Chinese lakes, such as Donghu Lake [18], Liangzi Lake [3], Dongting Lake [8], Yundang Lagoon [19], Dianchi Lake [20], Dalong Lake [21], and Taihu Lake [22]. The determination of TCS and BPA was mostly concentrated in southern China, while it was rare in the northern lakes.

Baiyang Lake is the largest freshwater shallow lake in North China, which is of great significance for regional climate regulation, ecological balance maintenance, and species diversity protection [13]. There are 36 water villages and 62 semi-water villages in Baiyang Lake. As a famous tourist destination in China, Baiyang Lake receives over 850,000 tourists annually. The anthropogenic inputs from household and tourism wastewater discharge would also result in a great increase in the pollutants into aquatic ecosystems [23]. In the past, the Baiyang Lake had eight rivers in its upper reaches. Currently, most of these rivers are experiencing seasonal droughts or have ceased to flow. The current stable supply rivers include Fu River and Baigouyin River. Fu River receives a large amount of wastewater from the sewage treatment plant in Baoding city. The Baigouyin River carries a large amount of sewage from sewage treatment plants, plush toys, food processing, and luggage industries in Baigou and Rongcheng counties [24]. Due to the urbanization and industrialization in recent decades, the water quality of the inflow river is also deteriorating, and the pollutants are commonly transferred to lakes through inflow rivers [23]. Since the establishment of Xiong’an New Area, Baiyang Lake has become an important ecological water source in the area. Therefore, ecotoxicology and human health risk assessments should be conducted to assess the collective potential environmental risks of multiple pollutants in order to ensure the safety of drinking water.

The occurrence and potential risks associated with parabens TCS and BPA in Baiyang Lake have not been documented thus far. Therefore, the surface water and sediment samples of Baiyang Lake were collected and evaluated in this study. The main goals were to: (1) analyze the occurrence and concentration of target compounds in surface water and sediment, (2) reveal the spatial distribution and the possible correlation of target compounds in surface water and sediment, (3) estimate the health risk of target compounds in surface water, and (4) estimate the ecological risk of target compounds in the surface water and sediment of Baiyang Lake. The results of this study could provide basic information for the pollution of target compounds and their human health and ecological risks in Baiyang Lake, which also provide scientific data support for the restoration plans of lake ecosystems.

## 2. Materials and Methods

### 2.1. Compounds and Materials

The n-hexane, dichloromethane, and acetone from Kaitong (Tianjin, China) were all HPLC grade; analytical pure anhydrous sodium sulfate was purchased from Kermel (Tianjin, China). All ultra-pure water was prepared using the Molelement 1810DF ultra-pure water system from Mole Water Treatment Equipment Co., Ltd. (Chongqing, China).

Chemical standards of methyl paraben (MeP, 99.7%), ethyl paraben (EtP, 98.3%), propyl paraben (PrP, 98.7%), butyl paraben (BuP, 99.5%), BPA (99.9%), and TCS (99.8%) were purchased from Anpel (Shanghai, China). Information on the structure and properties of the target personal care products is provided in Table S1 of the supplementary materials.

### 2.2. Study Area and Sampling

**Figure 1:**
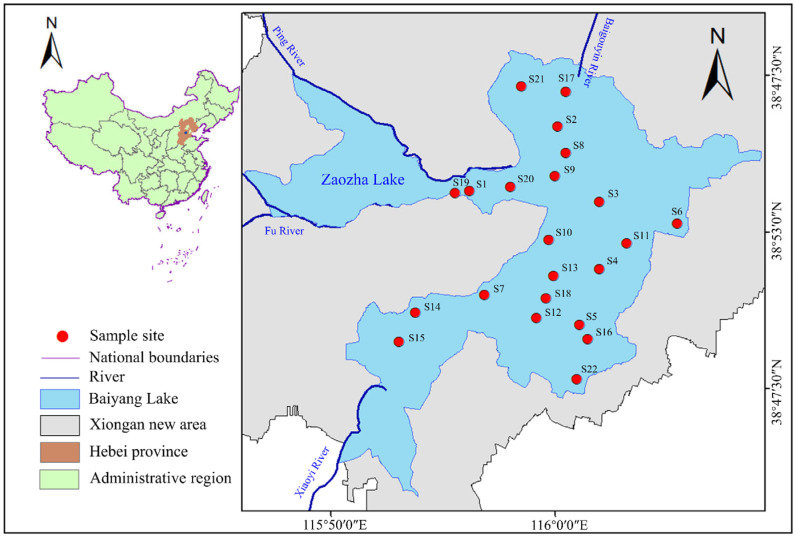
Map of sampling sites in Baiyang Lake.

Baiyang Lake is located in Xiong’an New Area, Hebei Province in China (39°04′–40°04′ N, 113°39′–116°11′ E), with a total water area of 366 km^2^. In September 2022, a total of 22 sites were selected in Baiyang Lake to investigate the parabens TCS and BPA in surface water and sediment, covering the entire lake except for the Zaozha Lake (Figure 1). Water and sediment samples were collected from the lake. All surface water samples were collected using an organic glass collector at 50 cm below the water’s surface, and then transferred into a 1 L brown glass bottle. When collecting water samples at each site, the corresponding bottles were pre-rinsed three times with sampled water. The collected water samples were then stored at 4 °C and pretreated within 48 h. Surface sediment samples were collected using a stainless-steel grab sampler, and then packaged in self-sealing bags covered with tin foil (soaked in dichloromethane for 24 h and dried before use). The bags were placed in an ice box and stored in the dark before being transported to the laboratory. Each sample was freeze-dried, grinded, passed through a 100-mesh copper sieve, and stored at −20 °C until analysis. Physical and chemical parameters such as pH, water temperature, and dissolved oxygen (DO) were measured using a portable multi-function water quality tester (Loveland, MA, USA) (Table S2).

### 2.3. Sample Pretreatment and Analysis

The slightly improved method of solid phase extraction (SPE) by Wan et al. (2018) [25] was used to extract the parabens TCS and BPA from water samples. Briefly, water samples and procedural blanks were filtered with a 0.45 μm glass fiber filter (Shanghai, China). The Oasis HLB cartridges for SPE were first conditioned with 6 mL n-hexane, 6 mL dichloromethane and 6 mL ultra-pure water. The water samples were then passed through the cartridges at a rate of 10 mL/min. After that, the cartridges were washed with 6 mL of ultra-pure water and dried for 0.5 h. Then, the analytes were eluted with 18 mL of a mixture of n-hexane and dichloromethane (v:v = 1:1) in three batches and rotated and evaporated to 1 mL. The concentrated solution was filtered through a 0.22 μm organic phase needle filter (Anpel, Shanghai, China) and transferred to a 2 mL glass sample vial. All samples were stored at −20 °C in a freezer until instrumental analysis.

Sediment samples were extracted using the ultrasound-assisted extraction followed by SPE clean-up method described by de Sousa et al. (2015) [26], with some modifications. Five grams of sediment was taken in a 50 mL glass tube, and then 25 mL of acetone was added and mixed in a vortex mixer and placed in an ultrasonic bath for 20 min at room temperature. Samples were centrifuged at 5000 rpm for 5 min, and the extraction solvent was transferred to another tube. The extraction was repeated two times. The combined extracts were purified with an Oasis HLB (6 mL, 200 mg; Rizhao, China), and then eluted with 18 mL of methanol three times. The eluent was rotated and evaporated to 1 mL before being subjected to analysis.

Target chemical compounds were analyzed with an Agilent 7890–5977A gas chromatography-mass spectrometer (GC–MS) (Santa Clara, CA, USA). The column temperature was set from an initial temperature of 50 °C for 1 min, ramped to 220 °C at a rate of 30 °C/min, held for 2 min, increased to 250 °C at 15 °C/min, and held for 5 min. Ultra-pure helium was used as the carrier gas for analysis at a flow rate of 1.2 mL/min with an HP-5 fused silica capillary column (15.0 m × 0.25 mm × 0.25 μm) (Folsom, CA). Selected ion monitoring (SIM) mode was applied for analysis, and the electron bombardment energy was 70 eV. The temperatures of the sample inlet and ion source were set to 280 °C and 300 °C, respectively.

### 2.4. Quality Assurance/Quality Control (QA/QC)

All collecting equipment, containers, and tin foil papers were rinsed with methanol and purified water to avoid sample contamination. The accuracy of the standard curve was verified using the target analytes standard mixture before each test. A procedural blank was set after every five samples to assess possible contamination during the experiment. In this study, individual target analytes were quantified using an external standard method. Six concentrations (0.1, 0.5, 1, 5, 10, and 20 μg/L) of standard solution were run to calculate calibration curves, and all the target analytes showed correlation coefficients higher than 0.995 (Table S3). The recoveries of these spiked target analytes in water and sediment samples were 76–126% and 67–116%, respectively, with relative standard deviations less than 10% (Table S3). Limit of detection (LOD) was measured as the concentration of the sample at a signal-to-noise ratio of 3, and limit of quantification (LOQ) was measured as the concentration of the sample at 10 times signal-to-noise ratio (Table S3).

### 2.5. Statistical Analysis

Descriptive statistics and normality tests were carried out using SPSS v26.0. A non-parametric Kruskal–Wallis test was performed to determine the significant difference in the concentrations of parabens TCS and BPA among sites (*p* < 0.05 to be statistically significant). A non-parametric Spearman’s correlation was performed to analyze correlations between parabens TCS and BPA and physicochemical parameters.

### 2.6. Risk Assessment

#### 2.6.1. Health Risk Assessment

Hazard quotient (HQ) is currently most commonly used to assess the human health risks of exposure to pollutants through a single pathway. The HQ to humans is generally determined by calculating the quotient of the estimated daily intake (EDI; ng/kg BW/d) and the acceptable daily intake (ADI; ng/kg BW/d):

(1) HQ=EDI/ADI×100%

(2) $$EDI=C_{s}\times IR_{w}/BW$$

where *C*_s_ is the concentration of target substances in drinking water, *IR*_w_ is the daily intake of drinking water, and BW is weight; specific values are shown in Table S4. In this study, the ADI of PrP was 0.1 mg/kg BW/d, the ADI value of the sum of MeP and EtP was 10 mg/kg BW/d, the ADI of BPA was 0.05 mg/kg BW/d, and TCS used 1.2 mg/kg BW/d. According to previous studies, HQ ≤ 1% indicates a negligible risk, 1% < HQ < 5% indicates a considerable risk, and HQ > 5% denotes a distinct risk for humans [27].

#### 2.6.2. Ecological Risk Assessment

In the present study, risk quotients (RQs) for three trophic levels (green algae, daphnia magna, and fish) were used to evaluate the potential ecological risks of parabens TCS and BPA in surface water and sediment. RQs were calculated by predicted or measured environmental concentration (PEC or MEC) divided by the predicted no-effect concentration (PNEC). In general, RQ’s calculation formula for target compounds in aquatic environments is as follows [21,28]:

(3) RQ=MEC/PNEC

(4) $$PNECwater=EC_{50}(LC_{50})/AF$$

where *MEC* is the measured concentration in the environment, *PNEC_water_* is the predicted ineffective concentration in the water, EC_50_ is the semi-maximum effective concentration, LC_50_ is the median lethal concentration, and AF is the evaluation factor. Toxicity data for this study were obtained from the USEPA Ecotoxicology database (USEPA, 2011) [29] (Table S5). Due to the current lack of toxicity data on target compounds in sediment, it is not possible to directly obtain the PNEC contents of target compounds in sediment from the literature. This PNEC conversion formula was used in this work to obtain the risk entropy value. The calculation formula is as follows [10]:

(5) $$PNEC_{sediment}=PNEC_{water}\times K_{d}$$

(6) $$K_{d}=K_{oc}\times F_{oc}$$

where *K_d_* is the sediment water distribution coefficient, *K_oc_* is the adsorption coefficient of organic compounds (Table S4), and *F_oc_* is the fraction of organic carbon in the corresponding sediment sample (Table S2). When 0.01 ≤ RQ < 0.10, it is considered low risk; 0.10 ≤ RQ < 1.00, indicates moderate risk; and RQ ≥ 1.00 indicates high risk.

## 3. Results and Discussion

### 3.1. Concentrations of Parabens TCS and BPA in Surface Water

The concentrations and detection frequencies of target compounds in the Baiyang Lake surface water are summarized in Table 1. Among them, MeP, TCS, and BPA were found in all the water samples, indicating the widespread use of these compounds in the watershed. EtP, PrP, and BuP were only detected in 59.1%, 68.2%, and 31.8% of the samples, respectively, which may be related to their relatively low neutral water solubility [28]. MeP was the dominant component of the parabens, followed by PrP. MeP represented the mean 74.2% of the total parabens in water. This composition may be consistent with their widespread use in many cosmetics and food in China [5]. The highest average concentration at 33.09 ng/L was found for BPA, followed by TCS, MeP, PrP, EtP, and BuP at 26.10, 2.07, 0.34, 0.30, and 0.08 ng/L, respectively. Over 206,000 tons of BPA are reportedly produced annually in China [30], while the total annual use of triclocarban and TCS was estimated to be 1220 tons based on the commodity consumption statistics [31]. The large-scale production and application of TCS and BPA, as well as their difficulty in being removed by traditional sewage treatment plants [1], may account for the widespread contamination of TCS and BPA in this region.

The comparison of the target compounds concentrations with those found in water samples from other lakes and wetlands around the world is summarized in Table S6. There were limited data about parabens in lakes from mainland China, and there has been no previous study on parabens in Baiyang Lake. Lower concentrations of parabens were found in our study than some other regions, including Sweden’s three largest lakes (n.d.–22 and n.d.–19 ng/L for MeP and PrP, respectively) [32], Lake Wierzbicza, Poland (7–38.4, 1.7–14.7, 3.7–10.4 and 1.3–5.5 ng/L for MeP, EtP, PrP and BuP, respectively) [33], Al-Hassa Shallow lakes, Saudi Arabia (n.d.–27.4, n.d.–6.2, n.d.–12.5 and n.d.–65.2 ng/L for MeP, EtP, PrP and BuP, respectively) [34], and Mediterranean coastal wetland, Spain (0–107, 0–82, 0–135 and 0–71 ng/L for MeP, EtP, PrP and BuP, respectively) [11]. This was probably due to the local government implementing a series of environmental governance and restoration projects, such as centralized treatment of domestic sewage from the perspective of watershed management, which could result in low concentrations of parabens; however, this needs to be confirmed in a future study. The concentrations of MeP and PrP observed in this study were similar with the previous reported concentrations in Lake Mälaren, Sweden (0.28–0.85 and 0.056–0.19 ng/L for MeP and PrP, respectively) [35].

Compared with the concentrations of TCS reported in other lakes, the concentration range of TCS in our study was similar to those reported in the Mediterranean coastal wetlands, Spain (0–72 ng/L) [11], while higher concentrations were reported in Lake Michigan, USA (max: 24 ng/L) [36], Al-Hassa shallow lakes, Saudi Arabia (n.d.–33.5 ng/L) [34], and Dongting Lake, China (mean: 10 ng/L) [8]. However, their concentrations were far lower than Donghu Lake (n.d.–466 ng/L) and Liangzi Lake (n.d.–239 ng/L) in Wuhan, China [3]. This may be due to increased use and emissions of PPCPs under the influence of urbanization, as well as severe COVID-19, leading to the accumulation of TCS in surface water in Wuhan [3].

Our study found a higher concentration of BPA than in Lake Wierzbiczańskie, Poland (6.7–19.5 ng/L) [33], Dongting Lake, China (5 ng/L) [8], Yundang Lagoon, China (14.2–31.4 ng/L) [19], Dalong Lake, China (15–53 ng/L) [21], Taihu Lake, China (19–68 ng/L) [22], and Bengaluru lakes, India (n.d.–26.4 ng/L) [17]. The concentration of BPA observed in this study was comparable to that reported in Venice Lagoon, Italy (<1.0–145 ng/L) [37], but lower than those reported in Al Hassa Shallow lakes, Saudi Arabian (n.d.–484.9 ng/L) [34], Mediterranean coastal land, Spain (12–205 ng/L) [11], and Dianchi Lake, China (15.5–530 ng/L) [20]. These results indicated that globally, the pollution levels of parabens in the surface water of Baiyang Lake were relatively low, while the pollution levels of TCS and BPA were at a moderate level.

### 3.2. Contents of Parabens TCS and BPA in Sediment

The minimum, maximum, mean, and detection rates of target compounds in sediment samples from Baiyang Lake are shown in Table 1. Six target substances were detected in sediment samples, and the detection rates were all higher than those of water. This is consistent with the observation in the Yellow River and the Huai River in Henan Province [5], and may be related to the logK_ow_ of six target compounds; the relatively high logK_ow_ (>1.9) makes them more inclined to accumulate in sediments. The contents of PBs were in the range of 2.42 to 18.37 ng/g. Like in surface water, MeP (2.42–13.56 ng/g) was still the main component of parabens in sediment, accounting for 77.05%, followed by PrP (n.d.–4.01 ng/g), EtP (n.d.–2.16 ng/g), and BuP (n.d.–0.17 ng/g). All sediment samples contained measurable TCS and BPA, with average contents of 24.5 and 32.54 ng/g, respectively.

The contents of parabens TCS and BPA in sediments of Baiyang Lake were compared with those from other regions in the world. The results are listed in Table S7. The contents of MeP in sediment samples in this study (2.42−13.56 ng/g) were comparable to those reported in sediment samples from Lake Shihwa, Korea (2.43−16.2 ng/g) [38] and Mediterranean coastal land, Spain (0–19 ng/g) [11], but higher than the levels found in Lake Mälaren, Sweden (<0.92–2.4 ng/g) [35]. We found that the contents of EtP, PrP, and BuP (n.d.−2.16, n.d.−4.01 and n.d.−0.17 ng/g for EtP, PrP, and BuP) were lower than some other regions, including the Mediterranean coastal wetland, Spain (ranges of EtP, PrP, and BuP: 0−18, 0−12 and 0−7 ng/g) [11] and Lake Shihwa, Korea (ranges of EtP, PrP, and BuP: 0.315−2.615, 0.097−64.5 and n.d.−29.1 ng/g) [38].

Compared with other report results (Table S7), the contents of TCS in sediments from Baiyang Lake (5.07−83.79 ng/g) were similar to those in Donghu Lake, China (n.d.–71 ng/g) [18], and exceeded those observed values in Liangzi Lake, China (n.d.–25 ng/g) [3] and Dongting Lake, China (mean: 4 ng/g) [8]. However, the contents of TCS were lower than Lake Michigan, USA (max: 150 ng/g) [36]. The contents of BPA (4.63−64.59 ng/g) were similar to those in Yundang Lagoon, China (21.2–50.9 ng/g) [19], exceeding the observed values of Dongting Lake, China (mean: 20 ng/g) [8] and Donghu Lake, China (n.d.–37.1 ng/g) [18]. However, they were much lower than those in Venice Lagoon, Italy (21.2–50.9 ng/g) [37], Anzali Wetland, Iran (21.2–50.9 ng/g) [39], and Dianchi Lake, China (21.2–50.9 ng/g) [20].

### 3.3. Spatial Distribution in Surface Water and Sediment

**Figure 2:**
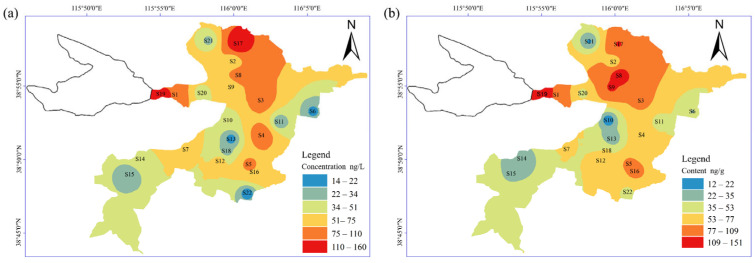
Spatial distribution of total parabens, TCS and BPA in surface water (**a**) and sediments (**b**).

**Figure 3:**
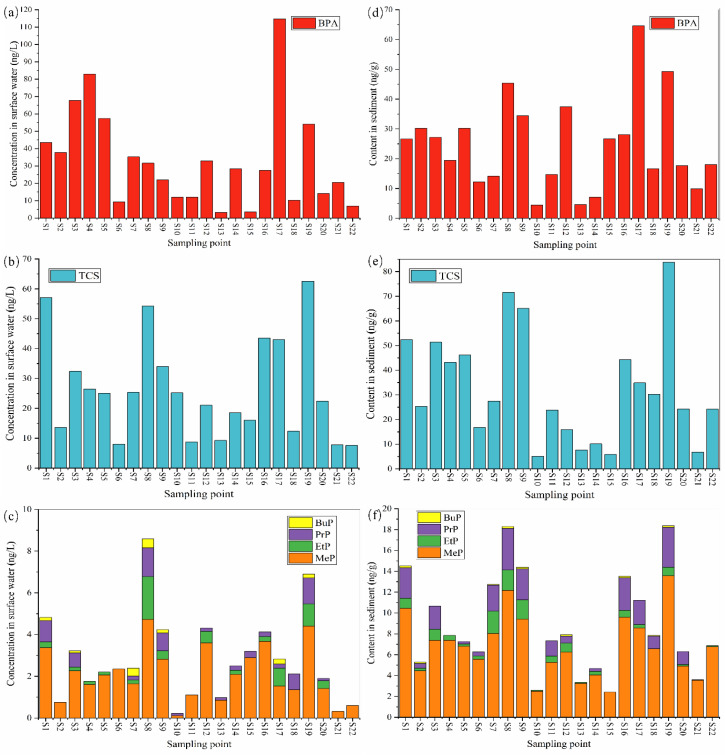
Spatial distribution of BPA (**a**,**d**), TCS (**b**,**e**), and parabens monomer (**c**,**f**) in surface water and sediments.

The spatial distribution of total concentrations and monomer concentrations of BPA, TCS, and parabens in water and sediment at different sampling sites are shown in Figure 2 and Figure 3, respectively. Significant differences were observed in the concentrations of BPA, TCS, and parabens among 22 sampling sites (*p* < 0.05). Spatially, the total concentrations of target compounds were significantly higher in water and sediment from the inlet and adjacent areas of the Fu River and Baigouyin River than that from the other areas of the lake (Figure 2). Specifically, the concentrations of BPA, TCS, and ∑PBs at S6, S13, S15, S21, and S22 sites were much lower than those in other places (Figure 3), which may be due to their distance from villages and estuaries, thus being less affected by human activities, while the highest concentration of BPA in water (114.74 ng/L; Figure 3a) and sediment (64.59 ng/g; Figure 3d) was found at site S17, which could be related to the Baigouyin River. The Baigouyin River has received the domestic wastewater and industrial wastewater of the upstream urban areas and industrial zones, and may result in a large amount of BPA inputs to this area [24]. The highest concentration of TCS (62.54 ng/L) was found in the water sample at site S19, followed by S1 (57.07 ng/L) (Figure 3b). This may be because the Fu River mainly receives the effluent from the sewage treatment plant in Baoding City, and the domestic sewage contains certain levels of TCS, but not all the TCS can be removed by the conventional wastewater treatment plant, resulting in TCS making its way into Fu River. Non-point source inputs from fluvial transport were important contributors for Baiyang Lake pollution in this area [40]. The highest content of TCS (83.79 ng/g) was also found in the sediment sample at site S19 (Figure 3e). High concentrations of TCS were also observed in water and sediment samples from some freshwater basins and coastal areas receiving municipal sewage, showing a similar spatial variation trend [3,8]. It is notable that S8 in water (54.19 ng/L) and sediment (71.59 ng/g) at S8 were also found to possess a relatively high concentration of TCS. Furthermore, the highest concentration of parabens in surface water and sediment (8.59 ng/L; 18.29 ng/g) was observed in S8 (Figure 3c,f), which was located in the village waterway of Guolikou, and there were many sewage pipelines nearby that might have pipeline leakage problems. The concentration of ∑PBs in the water at each site was relatively low. Several studies reported the hydrolysis of parabens can be affected by the pH of the surface water [5,41]. The pH value from samples in this study were weakly alkaline. In strong alkaline solutions, parabens hydrolyze to the corresponding carboxylic acid [41], and the hydrolysis of parabens in the aquatic environment might be not the main fate in this study area.

Spearman’s correlation analysis was conducted to verify the relationship among parabens TCS and BPA. As shown in Tables S8 and S9, MeP was positively correlated with PrP in surface water (r = 0.68, *p* < 0.05) and sediment (r = 0.80, *p* < 0.05), which is consistent with previously reported results in various environmental matrices, including surface water, food, indoor dust, sediment, and urine [28]. MeP and PrP were often used in combination to increase their antibacterial effects [5]. Moreover, a positive correlation was found among the concentrations of MeP, EtP, PrP, and BuP in both surface water and sediment (r = 0.46–0.80, *p*  <  0.01), indicating similarity in their sources in surface water. which is consistent with the result of parabens in Beijing [28]. TCS also had significantly positively correlated with MeP, EtP, PrP, BuP, and BPA in both surface water and sediment (r = 0.63–0.92, *p* < 0.05), indicating that these compounds might come from similar or related sources. However, there were positive correlations between EtP and BPA (r  =  0.59, *p*  <  0.05) in surface water, which were different to those of MeP, PrP, and BPA in sediment (r  =  0.66 and 0.58, respectively, *p*  <  0.05), suggesting that different sources existed in these two matrices.

### 3.4. Risk Assessment

#### 3.4.1. Health Risk Assessment

**Figure 4:**
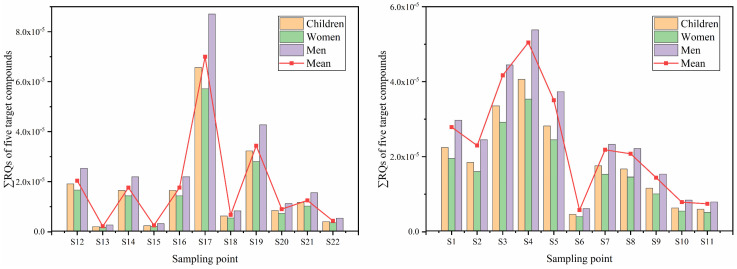
Overall and mean risk quotient of five target analytes in surface water at different sites for three groups of peoples.

The possible human health risks of parabens TCS and BPA in Baiyang Lake via drinking ingestion pathway were assessed by the methods described above. The health risk of BuP was not assessed, as it has no ADI value, so the health risks of only the remaining five target compounds were calculated for different populations in this study. First, we provide estimations of different populations of HQ based on the surface water concentrations of the target compounds in Baiyang Lake as a general condition of human exposure. After statistics, it was found that the HQs of all compounds were below 1, and these low values mean that the risk of all target compounds to human health can be ignored when considered separately (Table 2). BPA had the highest HQ value due to its lowest ADI value and higher detection concentration. On the contrary, EtP had the lowest HQ due to its relatively higher ADI values. It is worth noting that the HQs for children of all target compounds were higher than those of adults; therefore, more attention should be paid to the health risks of target compounds on children. Figure 4 shows the overall RQ of five target compounds in surface water at different sites. The results indicate that S17 had the highest average risk quotient value (7.0 × 10^−5^), followed by S4 and S3, and the average risk value at S13 (2.2 × 10^−6^) was the lowest. In general, the non-carcinogenic risk of the target compounds in water from Baiyang Lake did not cause harm to human health. However, the mass production and widespread use of parabens TCS and BPA will grow in various environmental media, and the health risks after long-term accumulation in water cannot be ignored. Therefore, a long-term human health risk assessment of such pollutants should be conducted in the future research.

#### 3.4.2. Ecological Risk Assessment

Parabens TCS and BPA may pose potential hazards to aquatic organisms according to previous reports [28]. The parabens TCS and BPA were frequently observed in water and sediment samples in this region. Therefore, it is essential to evaluate their ecological risks.

**Figure 5:**
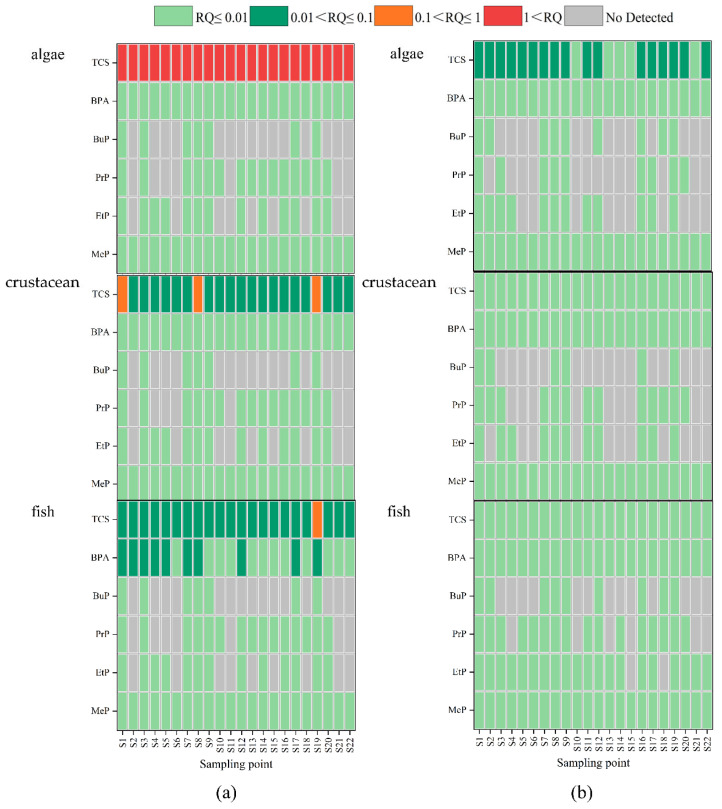
Ecological risk assessment of the target compounds at different trophic levels of aquatic organisms: algae, crustaceans, and fish in surface water, (**a**) and algae, crustaceans, and fish in sediment (**b**).

An ecological risk assessment of target compounds based on the chronic and acute toxicity of three main aquatic species (algae, invertebrates, and fish) is shown in Figure 5. The ecological risks of parabens and BPA to the three main aquatic species were negligible at most sites in Baiyang Lake. BPA was found to have low ecological risks to fish at ten stations in surface water (RQ > 0.1) (Figure 5). For example, Ramaswamy et al. [42] reported significant changes in the composition of phytoplankton communities in stream communities exposed to TCS concentrations as low as 15 ng/L, as well as a 33% reduction in algal richness exposed to 150 ng/L. It is worth noting that TCS presented a high ecological risk to algae in surface water and a low ecological risk in sediment (Figure 5a,b). As the main producer of the ecosystem, the ecological risks of algae were significantly higher than that of invertebrates and fish, which was consistent with previous research results [43]. Moreover, TCS generally presents low ecological risk to invertebrates and fish in most sites of surface water, but presents medium ecological risk to invertebrates and fish in three sites (S1, S8, and S19) and one site (S19), respectively. The high ecological risk level of TCS at S19 in surface water needs to be taken seriously, which also indicated that the input of the Fu River had a significant impact on Baiyang Lake. Although the ecological risks of parabens and BPA in this study area were not high, the mixed risks of all these compounds might still cause high levels of damage to aquatic communities.

## 4. Conclusions

In this study, the occurrence, distribution, and risk of parabens TCS and BPA were investigated in the surface water and sediment of Baiyang Lake. The results show that the main parabens were MeP and PrP; BPA and TCS were the main target pollutants in surface water and sediment, respectively. The concentrations of target compounds at the inlet of the Fu River and Baigouyin River in the upstream were significantly higher than that in the downstream. Parabens BPA and TCS are not likely to have adverse effects on human health at their current concentrations in the surface water and sediments of Baiyang Lake. Among all detected target compounds, TCS and BPA in surface water posed certain risks to three aquatic groups of organisms. In particular, TCS generally presents high risks to algae and medium risks to invertebrates and fish. Only TCS in sediment had low risks to algae. The results of the current study propose the implementation of the monitoring and management of rivers entering the lake, especially the Fu River and Baigouyin River. Since the effluent from the sewage treatment plant was an important supplement to the Fu River, investing in wastewater treatment is also recommended.

## Figures and Tables

**Table 1:** Concentrations and detection rates (DR) of target analytes in surface water and sediment from the Baiyang Lake.

Pollutant	Water (n = 22)				Sediment (n = 22)			
	DR (%)	Concentration (ng/L)			DR (%)	Content (ng/g)		
		Minimum	Maximum	Mean		Minimum	Maximum	Mean
MeP	100	0.11	4.72	2.07	100	2.42	13.56	6.77
EtP	59.1	n.d.	2.06	0.30	90.9	n.d.	2.16	0.61
PrP	68.2	n.d.	1.38	0.34	72.7	n.d.	4.01	1.35
BuP	31.8	n.d.	0.43	0.08	40.9	n.d.	0.17	0.05
TCS	100	7.59	62.54	26.10	100	5.07	83.79	32.54
BPA	100	3.24	114.74	33.09	100	4.63	64.59	24.50
∑PBs	–	0.22	8.57	2.79	–	2.42	18.37	8.78

n.d.: not detected.

**Table 2:** Human health risks of the target compounds for three groups of peoples in surface water.

Compound	HQ					
	Children		Women		Men	
	Mean	Maximum	Mean	Maximum	Mean	Maximum
MeP	1.5 × 10^−8^	3.5 × 10^−8^	1.0 × 10^−8^	2.3 × 10^−8^	1.2 × 10^−8^	2.7 × 10^−8^
EtP	2.3 × 10^−9^	1.5 × 10^−8^	1.5 × 10^−9^	1.0 × 10^−8^	1.7 × 10^−9^	6.0 × 10^−9^
PrP	1.3 × 10^−7^	5.1 × 10^−7^	8.4 × 10^−8^	3.4 × 10^−7^	9.6 × 10^−8^	3.9 × 10^−7^
BPA	2.5 × 10^−5^	8.6 × 10^−5^	1.6 × 10^−5^	5.6 × 10^−5^	1.9 × 10^−5^	6.5 × 10^−5^
TCS	8.1 × 10^−7^	1.9 × 10^−6^	5.3 × 10^−7^	1.3 × 10^−6^	6.1 × 10^−7^	1.5 × 10^−6^

## Data Availability

Data set is available upon request to corresponding authors.

## Supplementary Materials

The following supporting information can be downloaded at: https://www.mdpi.com/article/10.3390/toxics12010031/s1, Table S1: Physicochemical characteristics of the target compounds; Table S2: Water temperature, dissolved oxygen, and pH of surface water samples and fraction of organic carbon in sediments; Table S3: Analytical method validation parameters: linearity, recovery, precision, detection limit for target compounds in surface water and sediment; Table S4: Acceptable daily intake, adsorption coefficient of the target compounds and daily water intake, body weight of different populations; Table S5: Chronic or acute toxicity values and PNEC values of target compounds; Table S6: Comparison of the concentrations of parabens triclosan, and bisphenol A in surface waters from Baiyang Lake with those from other regions in the world; Table S7: Comparison of the contents of parabens triclosan, and bisphenol A in sediments from Baiyang Lake with those from other regions in the world; Table S8: Relationships of all the target compounds in surface waters; Table S9: Relationships of all the target compounds in sediments.
