# Supplementary material for: Parabens, Triclosan and Bisphenol A in Surface Waters and Sediments of Baiyang Lake, China: Occurrence, Distribution, and Potential Risk Assessment
Source: Toxics. 2023 Dec 31;12(1):31. doi: 10.3390/toxics12010031 (PMC10819025; doi:10.3390/toxics12010031)
Supplement: Supplementary file 1 [file toxics-12-00031-s001.zip › toxics-2749277-supplementary.pdf]

Supplementary Material

Parabens, Triclosan and Bisphenol A in Surface Waters and Sediments of Baiyang Lake, China: Occurrence, Distribution, and Potential Risk Assessment

Liguo Fu [a], Yaxue Sun [a], Hongbo Li [c], Yan Chen [a], Hui Du [a], Shu-xuan Liang [a, b, *]

[a] Key Laboratory of Analytical Science and Technology of Hebei Province, College of Chemistry and Materials Science, Hebei University, Baoding 071002, PR China.

[b] Institute of Life Science and Green Development, Hebei University, Baoding 071002, PR China

[c] Baiyangdian Basin Eco-environmental Support Center, Shijiazhuan 050056, China.

[*] Corresponding author

Shu-xuan Liang

E-mail address: liangsx168@126.com; Telephone number: +86-312-507-9359

Figure captions and Table legends

Table S1 Physicochemical characteristics of the target compounds.

Table S2 Temperature, dissolved oxygen (DO) and pH of surface water samples and fraction of organic carbon (Foc) in sediments.

Table S3 Correlation coefficient (R^2), recoveries (%, mean) from spiked matrices, relative standard deviation (RSD), limits of detection (LODs) and limits of quantitation (LOQs) for target compounds in surface water and sediment.

Table S4 Acceptable daily intake (ADI) and adsorption coefficient (K_{oc}) of the target compounds; daily water intake (IR_w) and body weight (BW) of different populations.

Table S5 Chronic or acute toxicity values and PNEC) values of target compounds.

Table S6 Comparison of parabens, triclosan and bisphenol A concentrations in surface water from the Baiyang Lake with those from other regions in the world.

Table S7 Comparison of parabens, triclosan and bisphenol A concentrations in sediment from the Baiyang Lake with those from other regions in the world.

Table S8 Relationship of all the targeted compounds in surface water.

Table S9 Relationship of all the targeted compounds in sediment.

Table S1. Physicochemical characteristics of the target compounds.

Compound	Structure	MW[a] (g/mol)	BP[b] (°C)	S_W[c] (mg/L)	pKa[d]	log Kow
Methyl paraben (MeP)	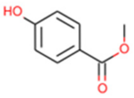	152.15	270	5981	8.17	1.96
Ethyl paraben (EtP)	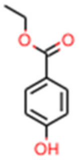	166.17	297.5	960	8.22	2.49
Propyl paraben (PrP)	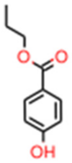	180.20	294.3	390	8.35	2.98
Butyl paraben (BuP)	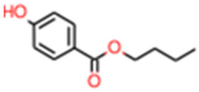	194.23	133	207	8.37	3.24
Triclosan (TCS)	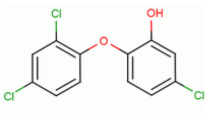	228.29	302.6	1200	7.9	10.29
Bisphenol A (BPA)	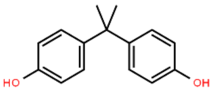	289.54	400.8	120	10.29	4.80

[a] MW: molecular weight; [b] BP: boiling point; [c] S_W: aqueous solubility at neutral pH; [d] pKa: acid-base reactivity.

Table S2. Temperature, dissolved oxygen (DO) and pH of surface water samples and fraction of organic carbon (Foc) in sediments.

Station	Longitude	Latitude	T (°C)	DO (mg/L)	pH	Foc (kg/g)
S1	115.9396	38.9063	12.6	4.76	6.8	0.066
S2	115.9980	38.9638	13.8	9.65	7.36	0.060
S3	116.0363	38.9011	14	5.49	7.55	0.085
S4	116.0370	38.8617	10	8.04	7.64	0.084
S5	116.0308	38.8226	11	9.65	7.53	0.079
S6	116.0949	38.8889	12	6.36	7.04	0.089
S7	115.9573	38.8415	10	9.72	7.65	0.106
S8	116.0111	38.9293	13.7	8.61	7.82	0.071
S9	115.9995	38.9171	13.6	7.58	7.92	0.070
S10	115.9991	38.8786	14.5	8.07	7.53	0.079
S11	116.0573	38.8772	10	9.73	7.74	0.091
S12	116.0082	38.8361	11	8.28	6.38	0.092
S13	116.0036	38.8575	9	7.69	7.71	0.090
S14	115.9001	38.8349	9	10.32	7.2	0.115
S15	115.8876	38.8176	10	9.47	7.3	0.106
S16	116.0284	38.8208	11	8.1	7.47	0.076
S17	116.0106	38.9651	13.8	9.93	7.52	0.060
S18	115.9971	38.8438	11	9.35	7.23	0.076
S19	115.9282	38.9051	13	6.55	7.76	0.066
S20	115.9698	38.9092	12.5	5.09	7.42	0.072
S21	115.9776	38.9679	13.7	10.49	7.64	0.060
S22	116.0210	38.7971	12.3	7.68	7.76	0.052

Table S3. Correlation coefficient (R^2), recoveries (%, mean) from spiked matrices, relative standard deviation (RSD), limits of detection (LODs) and limits of quantitation (LOQs) for target compounds in surface water and sediment.

Compound	R^2	Surface water				Sediment			
		Recoveries (%, mean)	RSD (%)	LOD (ng/L)	LOQ (ng/L)	Recoveries (%, mean)	RSD (%)	LODs (ng/g)	LOQs (ng/g)
MeP	0.9970	82.8	3.0	0.21	0.7	83.8	3.2	0.32	1.07
EtP	0.9990	85.2	3.5	0.12	0.4	83.4	5.8	0.26	0.86
PrP	0.9975	83.6	3.3	0.15	0.5	80.4	4.0	0.12	0.4
BuP	0.9969	81.2	2.8	0.03	0.1	69.7	3.1	0.07	0.23
TCS	0.9975	105	8.0	0.36	1.2	99	8.8	0.57	1.9
BAP	0.9952	114.2	7.7	0.39	1.3	101.4	5.9	0.63	2.09

Table S4. Acceptable daily intake (ADI) and adsorption coefficient (Koc) of the target compounds; daily water intake (IR_w) and body weight (BW) of different populations.

Compound	ADI (mg/kg BW/d)	Koc (L/kg)		IR_w (L/d)	BW (kg)
MeP	10	129	Children	1.000	26.800
EtP	10	246.9	Woman	1.350	55.100
PrP	0.1	510.3	Man	1.825	64.800
BuP	–	1002			
TCS	1.2	1245		.	
BPA	0.05	8417			

Table S5. Toxicity values and PNEC values of target compounds.

Analytes	Trophic level	Toxicity	Toxicity Value (mg/L)	AF [a]	PNEC (ng/L)
MeP	Algae	LC_{50}	50	1000	50000
	Invertebrate	EC_{50}	11.2	1000	11200
	Fish	EC_{50}	59.5	1000	59500
EtP	Algae	LC_{50}	18	1000	18000
	Invertebrate	EC_{50}	20	1000	20000
	Fish	EC_{50}	15	1000	15000
PrP	Algae	LC_{50}	36	1000	36000
	Invertebrate	LC_{50}	15.4	1000	15400
	Fish	EC_{50}	6.4	1000	6400
BuP	Algae	LC_{50}	0.058	1000	58
	Invertebrate	LOEC	0.2	100	2000
	Fish	EC_{50}	4.2	1000	4200
TCS	Algae	NOEC	0.0002	100	2
	Invertebrate	LC_{50}	0.47	1000	470
	Fish	LC_{50}	0.6	1000	600
BPA	Algae	NOEC	1.17	100	11700
	Invertebrate	NOEC	3.146	100	31460
	Fish	LC_{50}	3	1000	3000

[a] AF: assessment factor.

Table S6. Comparison of parabens, triclosan and bisphenol A concentrations in surface water from the Baiyang Lake with those from other regions in the world.

Location	Year	N[a]	Detected concentration (ng/L)						Region	Reference
			MeP	EtP	PrP	BuP	TCS	BPA		
Sweden's three largest lakes	2019−2020	51	n.d.[b] −22	−	n.d. −19	−	−	−	Sweden	Malnes et al. (2022)
Lake Mälaren	2017	7	<0.28−0.85	−	<0.056−0.19	−	−	−	Sweden	Golovko et al. (2020)
Lake Wierzbiczańskie	2015−2016	1	7−38.4	1.7−14.7	3.7−10.4	1.3−5.5	−	6.7−19.5	Poland	Czarczynska-Goslinska et al. (2017)
Al-Hassa shallow lakes	2017−2018	10	n.d. −27.4	n.d. −6.2	n.d. −12.5	n.d.−65.2	n.d.−33.5	n.d.−484.9	Saudi Arabia	Picó et al. (2020)
Mediterranean coastal wetland	2016−2017	32	0−107	0−82	0−135	0−71	0−72	12−205	Spain	Sadutto et al. (2021)
Lake Michigan	2009−2010	8	−	−	−	−	24[c]	−	USA	Blair et al. (2013)
Donghu Lake	2020−2021	8	−	−	−	−	n.d. −466	−	China	Wang et al. (2022)
Liangzi Lake	2020−2021	14	−	−	−	−	n.d. −239	−	China	Wang et al. (2022)
Donghu Lake	2013−2014	15	−	−	−	−	n.d. −6.5	n.d. −37.1	China	Wu et al. (2015)
Dongting Lake	2014	4	−	−	−	−	10[d]	5[d]	China	Xu et al. (2022)
Venice lagoon	2001−2002	4	−	−	−	−	−	<1.0−145	Italy	Pojana et al. (2007)

Yundang Lagoon	2008	4	–	–	–	–	–	14.2−31.4	China	Zhang et al. (2011)
Dianchi Lake	2011	10	–	–	–	–	–	15.5−530	China	Wang et al. (2012)
Dalong Lake	2015	5	–	–	–	–	–	15−53	China	Yu et al. (2016)
Taihu Lake	2016	26	–	–	–	–	–	19−68	China	Liu et al. (2017)
Bengaluru lakes	2018	6	–	–	–	–	–	n.d.−26.4	India	Gopal et al. (2021)
Baiyang Lake	2021	22	0.11−4.72	n.d. −2.06	n.d. −1.38	n.d.−0.43	7.6−62.54	3.2−114.74	China	This study

[a] N: number of sampling points; [b] n.d.: not detected; [c] max concentration; [d] mean concentration.

Table S7. Comparison of parabens, triclosan and bisphenol A concentrations in sediment from the Baiyang Lake with those from other regions in the world.

Location	Year	N[a]	Detected content (ng/g)						Region	Reference
			MeP	EtP	PrP	BuP	TCS	BPA		
Mediterranean coastal wetland	2016−2017	19	0−19	0−18	0−12	0−7	7−18	0−21	Spain	Sadutto et al. (2021)
Lake Shihwa	2008	34	2.43−16.2	0.315−2.67	0.097−64.5	n.d.[b]−29.1	−	−	Korea	Liao et al. (2013)
Lake Mälaren	2017	3	<0.92−2.4	−	−	−	−	−	Sweden	Golovko et al. (2020)
Liangzi Lake	2020−2021	14	−	−	−	−	n.d. −25	−	China	Wang et al. (2022)
Lake Michigan	2009−2010	8	−	−	−	−	150[c]	−	USA	Blair et al. (2013)
Dongting Lake	2014	5	−	−	−	−	4[d]	20[d]	China	Xu et al. (2022)
Donghu Lake	2013−2014	15	−	−	−	−	n.d. −71	n.d.−37.1	China	Wu et al. (2015)
Yundang Lagoon	2008	4	−	−	−	−	−	21.2−50.9	China	Zhang et al. (2011)
Venice lagoon	2000−2002	4	−	−	−	−	−	<2.0−118	Italy	Pojana et al. (2007)
Anzali Wetland	2010	5	−	−	−	−	−	10−6970	Iran	Mortazavi et al. (2012)
Dianchi Lake	2011	10	−	−	−	−	−	2.5−167	China	Wang et al. (2012)
Baiyang Lake	2021	22	2.42−13.56	n.d. −2.16	n.d. −4.01	n.d. −0.17	5.07−83.79	4.63−64.59	China	This study

[a] N: number of sampling points; [b] n.d.: not detected; [c] max concentration; [d] mean concentration.

Table S8. Relationship of all the targeted compounds in surface water.

	MeP	EtP	PrP	BuP	TCS	BPA
MeP	1.00					
EtP	0.70**	1.00				
PrP	0.68**	0.63**	1.00			
BuP	0.46*	0.68**	0.65**	1.00		
TCS	0.65**	0.81**	0.68**	0.71**	1.00	
BPA	0.31	0.59**	0.17	0.50*	0.68**	1.00

**The relationship is significant while $p < 0.01$

*The relationship is significant while $p < 0.05$

Table S9. Relationship of all the targeted compounds in sediment.

	MeP	EtP	PrP	BuP	TCS	BPA
MeP	1.00					
EtP	0.71^{**}	1.00				
PrP	0.80^{**}	0.75^{**}	1.00			
BuP	0.65^{**}	0.55^{**}	0.74^{**}	1.00		
TCS	0.92^{**}	0.63^{**}	0.76^{**}	0.64^{**}	1.00	
BPA	0.66^{**}	0.41	0.58^{**}	0.53^{*}	0.69^{**}	1.00

**The relationship is significant while $p < 0.01$

*The relationship is significant while $p < 0.05$
